# The Cost of Control: Financial and Administrative Burdens of Limited Distribution Network Changes in Cystic Fibrosis

**DOI:** 10.1002/ppul.71829

**Published:** 2026-09-14

**Authors:** Brittany A. Wright, Cameron J. McKinzie, Charissa W. Kam, Casey R. Tak, Lynnsey M. Knockel, Julie Langhorst, Rebecca S. Pettit, Lynda Roe, Billie Simonson, Hilary Vogt, Nicole Willer, David C. Young

**Affiliations:** ^1^ University of California San Diego La Jolla California USA; ^2^ University of Iowa Health Care Iowa City Iowa USA; ^3^ UNC Health Chapel Hill North Carolina USA; ^4^ University of Utah Salt Lake City Utah USA; ^5^ Indiana University Health Indianapolis Indiana USA; ^6^ Riley Hospital for Children at IU Health Indianapolis Indiana USA; ^7^ University of Utah Health Salt Lake City Utah USA; ^8^ University of Utah Adult Cystic Fibrosis & Asthma Centers Salt Lake City Utah USA

**Keywords:** CFTR modulators, medication access and affordability, pharmacy services

## Abstract

**Background:**

The clinical impact of pharmacists and pharmacy technicians on the care of people with cystic fibrosis (pwCF) has been well documented within the literature, including medication access, reduction of hospitalizations, and consolidation of pharmaceutical delivery for pwCF with complex medication regimens. However, challenges related to access and affordability have become increasingly complex, increasing the administrative burden placed upon these members of the multidisciplinary CF care team. The goal of this study was to quantify the time pharmacists and pharmacy technicians at CF Foundation‐accredited care centers (CFACC) spent triaging and resolving errors, categorize the types of interventions required, and tabulate the financial burden for pwCF averted through these interventions.

**Methods:**

This study was a retrospective review of pharmacy team members' interactions following a change in limited distribution network of cystic fibrosis transmembrane conductance regulator modulators (CFTRm) over a 4‐month period of time. Each site received institutional review board exemption. Measures were characterized using descriptive statistics and were analyzed in SAS v9.4 (Cary, NC). Collected data were extrapolated to be nationally representative based on the 2023 CFF Patient Registry report.

**Results:**

Six CFACC and 1781 pwCF were included in this review. Of these pwCF, 216 required additional interventions following a required change in the dispensing pharmacy for their CFTRm. Collectively, 201.5 h were spent on resolution of issues during the study period with interventions successfully averting a financial burden to pwCF of $226,094.73. Extrapolations of time and financial burden were estimated to be 3700 h and over $4.2 million, respectively.

**Conclusions:**

Medication access and affordability continue to present significant barriers for pwCF. We aim to highlight the critical services provided by clinical pharmacy teams at CFACC as well as their integrated health‐system specialty pharmacies, and the vital role they play in improving outcomes and providing cost‐effective care for pwCF.

## 1 Background

The clinical impact of pharmacists and pharmacy technicians on the care of people with cystic fibrosis (pwCF) has been well documented within the literature [1, 2, 3, 4]. Similarly, cystic fibrosis (CF) care centers with integrated health system specialty pharmacies (IHSSP) and pharmacy teams have shown to improve medication access, reduce hospitalizations, and consolidate pharmaceutical delivery for pwCF with complex medication regimens by streamlining prescribing, prior authorization approvals, and medication delivery [5, 6, 7, 8, 9, 10]. These positive impacts on patient care led to a revision in the CF care team model in November 2024, which now requires pharmacists as core members of the multidisciplinary CF care team and pharmacy technicians as programmatic components [11].

Given the development of highly‐effective cystic fibrosis transmembrane conductance regulator modulators (CFTRm) and the dramatic change of CF care for those pwCF eligible, including reduced hospitalizations and exacerbations, improved nutritional support, and overall extended life expectancy and quality of life, the role of clinical pharmacy services within CF care centers has shifted to have a greater emphasis on outpatient responsibilities [2, 12, 13]. With increasing usage of high‐cost therapies, concerns regarding medication access and affordability have become increasingly prominent. Unfortunately, this continues to expand the administrative burden to these members of the multidisciplinary CF care team, irrespective of where the patient is filling their medications [14, 15]. In a published study by Shaw et al. a reported $3.2 million of uncompensated specialty pharmacy work was completed by one IHSSP in order to ensure medication access and care coordination for their patients who were mandated to fill at external specialty pharmacies [16].

In November 2024, the manufacturer of CFTRm implemented a significant change to access by modifying its limited distribution pharmacy network. As a result, pwCF may have been required to transfer their CFTRm prescriptions from their IHSSP to a large commercial specialty pharmacy. The goal of this study was to quantify the time pharmacists and pharmacy technicians at CF Foundation‐accredited care centers (CFACC) spent triaging and resolving errors related to this change, categorize the types of interventions required, and tabulate the financial burden for pwCF averted through these interventions.

## 2 Methods

This multi‐center study was a retrospective review of pharmacy team members' interactions during the CFTRm change in limited distribution network of pharmacies, from November 1, 2024 through February 28, 2025. We collected data from six CFACC (three pediatric and three adult), representing a geographically diverse patient population from the US Southeast, Midwest, and West. Each site's institutional review board (University of Iowa, University of North Carolina, Indiana University, and University of Utah) independently reviewed for the proposal for approval or exemption. All sites granted exemption status and declined the need for informed consent due to lack of human subject involvement.

The following information was obtained for each encounter requiring intervention(s) from a pharmacist or pharmacy technician: role of the team member involved in the intervention (e.g., pharmacist, pharmacy technician), type of intervention (e.g., prescription/therapy clarification, prior authorization, billing/copay coordination, order status, billing issue, fielding a complaint, other), time invested to resolve the encounter issue (estimated to the nearest 15 min increment and capped at > 60 min), and resultant changes in billing charges (in then‐current US dollars), as applicable. Measures of frequency and types of interventions, the time to resolve the errors, and the billing charges averted were characterized using descriptive statistics and were analyzed in SAS v9.4 (SAS Institute, Cary, NC). Collected data were extrapolated to be nationally representative based on relative size of sample to 33,288 pwCF in the US based on the 2023 CFF Patient Registry report [17].

## 3 Results

**Figure 1:**
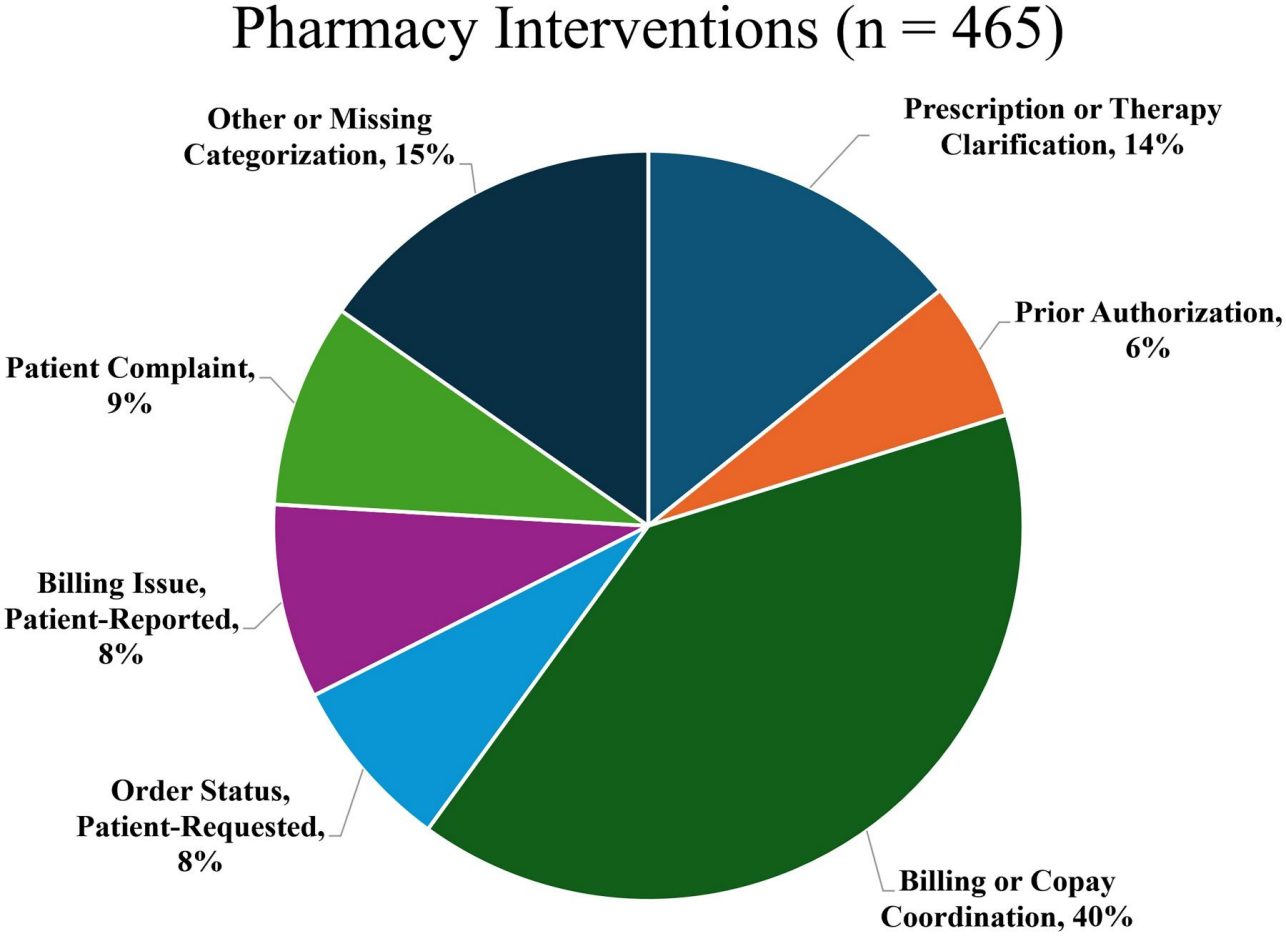
Interventions completed by pharmacists and pharmacy technicians at CF Foundation‐accredited care centers following a change in limited distribution network. [Color figure can be viewed at wileyonlinelibrary.com]

Six CFACC were included in the analysis. In total, these centers provide care to 1781 pwCF. Of these pwCF, 216 individuals required a total of 465 (mean: 2.2 per individual; standard deviation: 1.7) additional interventions following a required change in the dispensing pharmacy for their CFTRm. Pharmacists provided support in 45.4% (*n* = 211) of interventions and pharmacy technicians aided with 54.6% (*n* = 254) of interventions. Collectively, 12,090 min or 201.5 h were spent on resolution of issues during the study period (median: 37.5 min; range: 0–382.5 min). Most issues (55.6%) required multiple interactions to reach resolution (median: 2; range: 1–19). The most common interventions included billing or copay coordination (*n* = 185; 39.8%), prescription or therapy clarification (*n* = 66; 14.2%), or prior authorization status (*n* = 28; 6.0%). Median time spent on billing or copay coordination, prescription or therapy clarification, and prior authorization status was 15 min, 22.5 min, and 22.5 min, respectively. Additional time and cost analysis of individual intervention type is noted in Table 1. Complaints, billing issues, and order status requests by pwCF or their caregivers accounted for another 25% of pharmacy team interventions and 15% (*n* = 71) of the interventions were indicated as other or missing categorization. These interventions are represented in Figure 1. At least 13 of these identified access issues resulted in a known lapse of CFTRm therapy for pwCF.

**Table 1:** Time and patient medication cost averted per intervention category.

Intervention Category	Variable	*N*	Minimum	Mean	Standard Deviation	Median	Maximum
Billing or copay assistance	Time, m	185	0	32.1	22.5	22.5	120
	Cost, $	33	40	5076.95	2250.79	5900.00	9000.00
Prescription or therapy clarification	Time, m	66	15	21.6	17.0	15	97.5
	Cost, $	1	6000	6000		6,000	6000
Prior authorization status	Time, m	28	15	35.4	21.8	22.5	97.5
	Cost, $	4	3500	4900.16	1036.06	5050.31	6000

Abbbreviations: m, minutes; N; number; $, US dollars.

For the 41 (19.0%) pwCF who had a monetary amount linked to the intervention, the CFACC clinical pharmacy team's work successfully averted a total financial burden to pwCF of $226,094.73 over the study period (per patient median: $5900; range: $40.00– $16,019.46).

Although this study represents data from just six centers, if extrapolated to the broader CF community in the US and assuming similar rates of interventions regardless of pharmacy support available, the projected impact would be 3751.4 h of pharmacist and pharmacy technician effort and an estimated financial burden averted of over $4.2 million during the 4‐month study period. Observed data and extrapolations are also shown in Table 1.

## 4 Discussion

Medication access and affordability continue to present significant barriers for pwCF. Pharmacists and pharmacy technicians, particularly those integrated into their CFACC multidisciplinary care teams, are well‐positioned to assist with these concerns and serve as a liaison between pwCF, other members of the multidisciplinary CF care team, pharmacies, pharmacy benefit managers, and health insurance companies. Our study results underscore the impact these team members have on pwCF. Nearly 40% of interventions provided by pharmacists and pharmacy technicians included billing and copay coordination. This included ensuring appropriate billing to primary or secondary insurance plans, obtaining manufacturer copay assistance programs, and correct utilization of tertiary grants. These interventions are only possible because pharmacy team members continue to devote substantial time to understanding insurance structures, how multiple insurances coordinate benefits, thorough knowledge and deployment of copay programs, as well as availability and correct processing of co‐pay assistance grant funds.

Additionally, 14.2% of interventions by pharmacy team members included prescription and therapy clarification. It is important to note that most of these clarifications were related to information that was already documented on the electronic prescription before being sent to the pharmacy; thereby adding an additional, unnecessary step of intervention and introducing a potential risk of therapy lapse due to delayed processing. While we acknowledge that 6% pwCF experienced a lapse in CFTRm therapy, it should be noted that 94% of pwCF didn't experience any lapse in therapy due to the swift and timely action of pharmacists and pharmacy technicians. This ability to triage and handle medication‐related access efficiently is a hallmark of dedicated pharmacy support.

Lastly, 6% of interventions included requests for prior authorization data. Of note, through the previously discussed IHSSP model, most pharmacists and pharmacy technicians verify benefits and obtain medication coverage prior to sending a prescription externally to ensure no further documentation is needed and improve transitionary care for the person with CF. This coordinated effort and process has been shown to reduce the time from medication prescribing to approval and medication initiation; therefore, these additional requests may add another point of vulnerability for medication delay [5, 7].

The CFACC clinical pharmacy team's interventions during the study period successfully averted a financial burden to pwCF of over $226,000. This included inappropriate billing to insurance, incorrect copay assistance tier based on insurance structure, incorrect billing to third‐party grants, or, unfortunately, quoted medication costs to pwCF. Correcting these errors as a third party rather than through the processing pharmacy adds time and effort because external pharmacies lack the transparency for care teams that is found with an IHSSP; however, these corrections are still completed by clinical pharmacy teams to ensure optimal and affordable care for pwCF. Unfortunately, these processing errors also resulted in several incidences of lapses of medication therapy. Lapses of therapy with other CFTR modulators have resulted in development of withdrawal symptoms and clinical decline; therefore, should not be underestimated [18].

The CFACC included in this study are large but are in no way all‐encompassing of the entire CF community. Given that all CFACC were impacted by this change, if our time and medication cost savings data were extrapolated to the larger CF community (33,288 pwCF per the 2023 CFF Patient Registry), an estimated 3700 h of pharmacist and pharmacy technician effort would have been required to complete these interventions and an estimated financial burden averted of over $4.2 million would have resulted from this change to the limited distribution pharmacy network. Notably, these figures are representative of the limited 4‐month study period and do not account for the ongoing efforts of pharmacists and pharmacy technicians for these medications, nor do they reflect time spent on non‐CFTRm issues. While these six CFACC are fortunate to have robust pharmacy involvement, this varies substantially across centers. Currently, there remain CFACC without pharmacy support, raising additional concerns about how a substantial change like the one in this study would be navigated. Without knowledgeable, integrated pharmacy support, these responsibilities will inevitably revert to pwCF and other members of the multidisciplinary CF care team—or risk not being identified at all.

It is worth pointing out that there are programs in place that attempt to bridge these gaps and coordinate between pwCF, care teams, and pharmacies, such as manufacturer liaison personnel and disease state‐specific concierge contacts. However, these external resources are limited in their efficacy for several reasons. First, they lack real‐time health information about individual pwCF, which leads to inaccurate or potentially harmful information being communicated. Second, the external nature of these resources creates unnecessary delays based on the increased time needed for communication between different stakeholders throughout the process. Lastly, and most importantly, these external resources represent unknowns for pwCF and their families. They lack the years of trust built in the relationship between pwCF and CFACC. Conversely, pharmacists and pharmacy technicians integrated within CFACC and IHSSP, like those included in this study, offer improved communication and transparency to pivotal parties, availability of the most up‐to‐date health information through the electronic medical record, and most importantly, earned patient trust.

As the medication access landscape continues to evolve and present new challenges for pwCF and their CF care teams, pharmacy team members will continue to provide vital support to remove and mitigate these barriers to ensure continued medication access. Manufacturers with limited distribution networks are encouraged to consider the widespread impacts of changes to these programs on pwCF, CF care teams, and the broader CF community, with the goal of making changes that lessen, rather than increase, barriers to care.

## Author Contributions

**Brittany A. Wright:** conceptualization, investigation, writing – original draft, methodology, writing – review and editing. **Cameron J. McKinzie:** conceptualization, methodology, writing – review and editing, investigation. **Charissa W. Kam:** investigation, writing – review and editing, conceptualization, methodology. **Casey R. Tak:** formal analysis, data curation, writing – review and editing. **Lynnsey M. Knockel:** writing – review and editing, investigation. **Julie Langhorst:** investigation, writing – review and editing. **Rebecca S. Pettit:** investigation, writing – review and editing. **Lynda Roe:** investigation, writing – review and editing. **Billie Simonson:** investigation, writing – review and editing. **Hilary Vogt:** investigation, writing – review and editing. **Nicole Willer:** investigation, writing – review and editing. **David C. Young:** investigation, writing – review and editing.

## Funding

The authors have nothing to report.

## Conflicts of Interest

Cameron J. McKinzie reports one‐time of service on a Vertex advisory board and has received grant funding from the Cystic Fibrosis Foundation.

## Data Availability

The authors have nothing to report.
